# Mental stress objective screening for workers using urinary neurotransmitters

**DOI:** 10.1371/journal.pone.0287613

**Published:** 2023-09-08

**Authors:** Kazuhiro Tanabe, Asaka Yokota

**Affiliations:** 1 Medical Solution Promotion Department, Medical Solution Segment, LSI Medience Corporation, Tokyo, Japan; 2 Kyushu Pro Search Limited Liability Partnership, Fukuoka, Japan; Sohag University Faculty of Medicine, EGYPT

## Abstract

**Background:**

Almost 10% of the population develop depression or anxiety disorder during their lifetime. Considering that people who are exposed to high stress are more likely to develop mental disorders, it is important to detect and remove mental stress before depression or anxiety disorder develops. We aimed to develop an objective screening test that quantifies mental stress in workers so that they can recognize and remove it before the disorder develops.

**Methods:**

We obtained urine specimens from 100 healthy volunteers (49 men and 51 women; age = 48.2 ± 10.8 years) after they received medical checks and answered the Brief Job Stress Questionnaire (BJSQ). Participants were divided into high- and low- stress groups according to their total BJSQ scores. We further analyzed six urinary neurotransmitters (dopamine, serotonin, 5-hydoroxyindoleacetic acid, gamma-aminobutyric acid, homovanillic acid, and vanillylmandelic acid) using liquid chromatography-mass spectrometry to compare their levels between the two groups.

**Results:**

We obtained the concentrations of the six analytes from 100 examinees and revealed that the levels of urinary dopamine (*p* = 0.0042) and homovanillic acid (*p* = 0.020) were significantly lower in the high-stress group than those in the low-stress group. No biases were observed between the two groups in 36 laboratory items. The stress index generated from the six neurotransmitter concentrations recognized high-stress group significantly. Moreover, we discovered that the level of each urinary neurotransmitter changed depending on various stress factors, such as dissatisfaction, physical fatigue, stomach and intestine problems, poor appetite, poor working environments, sleep disturbance, isolation, worry, or insecurity.

**Conclusion:**

We revealed that urinary neurotransmitters could be a promising indicator to determine underlying mental stress. This study provides clues for scientists to develop a screening test not only for workers but also for patients with depression.

## Introduction

Approximately 2–15% of the population experiences depression or anxiety disorders at some point in their lives [1–3]. However, diagnosing these disorders still relies on subjective assessments by doctors, making it challenging to identify pre-disease states with an increased risk of developing these disorders. Several studies have explored biomarkers for diagnosing mental disorders, including major depressive disorder [4–6], panic disorder [7, 8], postpartum depression [9–11], post-traumatic stress disorder [12–14], autism [15], and adolescent anxiety disorder [16, 17]. Yet, the evidence supporting the use of these biomarkers for diagnosis remains insufficient [5]. Detecting and addressing mental stress before it develops into a disorder is crucial, as high levels of stress increase the risk of mental disorders [18]. Cortisol is a reliable biomarker for measuring stress levels [19], but its measurement requires strict control of sampling timing due to circadian fluctuations [20]. Neurotransmitters have been found to respond to mental states, with dopamine (DA) influencing motivation control [21, 22], gamma-aminobutyric acid (GABA) playing a role in reducing neuronal excitability [23], serotonin (5-HT) regulating mood, appetite, and sleep [24], and urinary homovanillic acid (HVA) and vanillylmandelic acid (VMA) used as neuroblastoma biomarkers [25, 26]. Furthermore, their levels in urine are reported to decrease in individuals with depression [24–27]. While serum or plasma are commonly used in medical tests, they may not always be ideal due to limitations in sampling locations, primarily restricted to medical facilities like hospitals or clinics. Given that the aim of this test is to identify mental stress in healthy individuals, sampling opportunities should be made more widely available, such as in companies or schools where access to clinical experts may be limited. In this context, non-invasive sampling methods like urine or saliva would be ideal for this test, as they offer greater convenience and ease of collection.

Developing a new screening test poses challenges, particularly in accurately identifying and recruiting examinees with asymptomatic mental stress. Traditional self-report questionnaires, such as the Beck Depression Inventory [28], Patient Health Questionnaire-9 [29], and Center for Epidemiologic Studies Depression Scale (CES-D) [30], are limited in identifying underlying mental stress in healthy individuals as they were designed to confirm depression or anxiety disorders. In contrast, the Brief Job Stress Questionnaire (BJSQ), developed by the Japanese government, assesses not only mental stress but also employees’ work environments and personal lives, making it more sensitive to mild mental stress and underlying stress among healthy individuals [31, 32].

In Japan, employees often face expectations of long working hours, placing limitations on personal time. The emphasis on teamwork and consensus-building often creates mental pressure when individual needs are subordinated to group interests. Additionally, the traditional lifetime employment system, still present in certain sectors, restricts employee mobility and sometimes keeps employees under stressful conditions. Recognizing work-related stress as a major factor in overwork-related deaths, the government implemented the BJSQ in all companies with more than 50 employees since 2015.

The BJSQ evaluates four key factors: (1) direct stressors in the work environment, such as overwork and interpersonal relationships; (2) coping ability, including problem-solving skills and ability to navigate challenging situations; (3) coping resources, such as social communication skills and support from family or colleagues; and (4) physical disorders, including conditions like depression or insomnia [31, 32]. Hence, our study defines stress based on these interconnected factors, providing a comprehensive assessment of work-related stressors and their impact on individuals.

Although urinary neurotransmitter levels may not always reflect those in the brain, we hypothesize that they can still serve as potential biomarkers for monitoring mental health conditions and assessing the degree of mental stress. In this study, we aim to establish a robust and reliable screening test using urine samples from healthy volunteers, targeting six neurotransmitters (DA, 5-HT, 5-HIAA, GABA, HVA, and VMA).

## Methods

### Study design and recruiting participants

**Fig 1:**
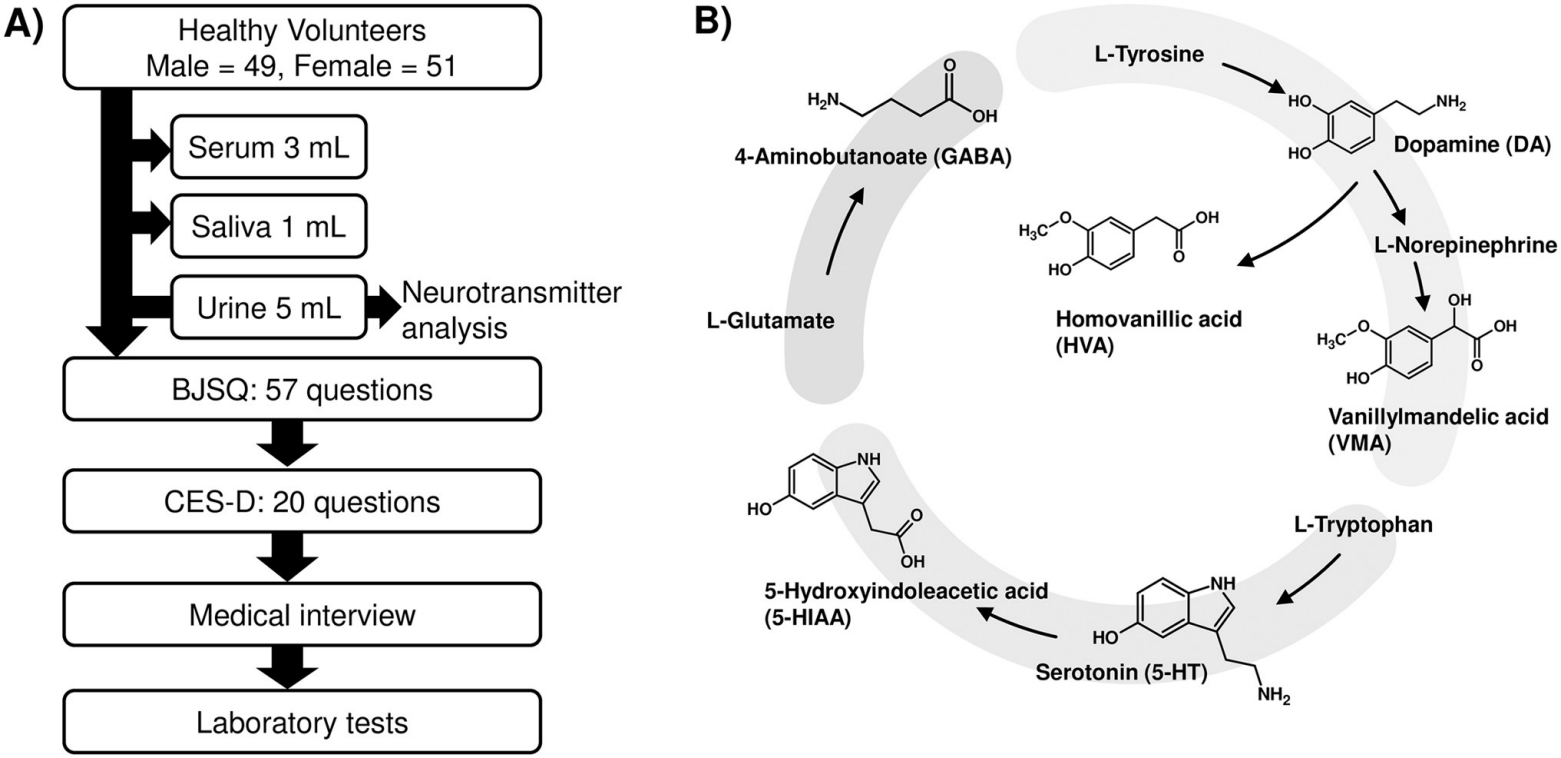
Study design and target analytes. This study was conducted following the scheme shown in A). All participants started fasting from 9 p.m. and sampling was conducted the next morning. Serum, urine, and saliva were obtained from all participants. All participants answered BJSQ and CES-D items and were interviewed by a medical doctor. All participants received laboratory tests. The target urinary neurotransmitters are shown in B) with their metabolism pathway.

All participants were recruited by Soiken Inc. The Institutional Review Board of Fukuda Clinic approved this study on June 15, 2019 (no. IRB-20190615-3) for the use of patients’ clinical information and specimens. We assumed that the average concentration of neural transmitters would differ by no more than half of the standard deviations within each group. Based on this assumption, we estimated a sample size of approximately 50, considering an alpha-error (0.05) and beta-error (0.2) framework. Participants were recruited from July 1 to 31, 2019 and written informed consents were obtained from all patients. All participants started fasting from 9 p.m. and sampling and self-report questionnaires were conducted in the next morning. Serum, urine, and saliva and subjects’ data were obtained from all participants during the above-mentioned period; however, only urine was used in this study. Authors cannot access to information that can identify individual participants during and after data collection. All participants answered the BJSQ (S1 Text), provided by the Ministry of Health, Labour and Welfare in Japan. The CES-D (S2 Text) was also administered to all participants to check the accuracy of BJSQ answers (Fig 1A). The BJSQ comprises 57 items and the CES-D comprises 20 items. Participants chose one of four options depending on their mental conditions. Since both the BJSQ and CES-D include items that ask about both positive (e.g., “I felt hopeful about the future”) and negative (e.g., “I felt depressed”) feelings, we reversed the scores of the positive items before summing all scores. We did not sum up scores of some items which the participants did not answer.

Participants were interviewed by medical doctors, which included items about smoking and alcohol drinking habits and their familial health histories. Participants who were judged as unhealthy by the doctor (e.g., serious renal failure, hepatic failure, diabetes, etc.) were excluded. Finally, participants underwent laboratory tests including 36 items related to hepatic, renal, and metabolic functions.

### Target analytes and reagents

The six analytes and their relationships are shown in Fig 1B with their metabolic pathways. Standard chemicals of 5-HIAA, DA, and VMA were purchased from TCI (Tokyo, Japan). Those of GABA, 5-HIAA, HMA, and creatinine (Cre) were obtained from Sigma (MO, USA). Internal standards, such as DA-d4, GABA-d6, 5-HT-d4, HVA-d3, VMA-d3, and CR-d3 were purchased from TCI. The analytical-reagent grade was selected for the other chemical reagents.

### Preparation

Urine samples (10 μL), water (230 μL), and internal standard solution (10 μL) were added to a 1.5-mL microtube and mixed with a vortex mixer. The working solution (10 μL) was added instead of the urine sample when the standard curve was created. The working solution for the standard curves was prepared by mixing six neurotransmitters at 100 μg/mL each with solution A (50% methanol, 3% acetic acid, and 47% water (v/v/v)). The solution was diluted to 30, 10, 1, 0.3, 0.1, 0.03, and 0.01 μg/mL with solution A. Internal standard solution was prepared by mixing DA-d4, GABA-d6, 5-HT-d4, HVA-d3, VMA-d3, and CR-d3 at 10 μg/mL each with solution A. 5-HT-d4 was also used for the quantification of 5-HIAA because deuterium-labeled 5-HIAA could not be commercially obtained. The mixed solution was added to an ultrafiltration tube (Amicon 30 K, Millipore Corp., MA, USA) and centrifuged at 10,000 g at room temperature for 30 min to remove proteins. Since the levels of six neurotransmitters are quite different, we prepared two diluted solutions from one sample (non-dilution and 100-fold dilution) to analyze both low- and high-concentration biomarkers simultaneously.

### LC-MS

We developed an analytical method based on several articles [33–37]. The six neurotransmitters and CR were analyzed by a LC-MS system (Agilent ULTIVO, Agilent Technologies; Santa Clara, CA, USA) equipped with a Shim-pack MAqC-ODS I column (2.1 mm × 150 mm 2.7 μm; Shimadzu, Kyoto, Japan). Mobile phase A buffer was 0.1% formic acid in water, and B buffer was 0.05% formic acid in methanol. The neurotransmitters were eluted at a flow rate of 0.2 mL/min at 40°C with the following gradient program: 1% B buffer (0 to 0.5 min), 1% to 10% B buffer (0.5 to 4.0 min), 10% to 50% B buffer (4.0 to 10.0 min), and 3 min hold at 100% B buffer. Before starting the next run, the initial condition was held for 2 min (15 min per run). The injection volume was 5.0 μL and the autosampler temperature was maintained at 4°C. Ionization of each analyte was achieved using electrospray ionization, and the conditions of polarities (positive or negative), precursors, products, and collision energies were determined as shown in S1 Table to maximize their peak intensities. Quality control (QC) was prepared by pooling urines of 18 healthy volunteers and dividing them into 10 μL each in a 1.5-mL microtube. They were stored at -80°C until analysis. QC and a blank were analyzed along with the calibration curve at the start and end of each run. When the values of QC were within 15% of their predicted values, the run was regarded as validated.

### Data analysis

Data analysis was performed using the Masshunter software package (Agilent Technologies; Santa Clara, CA, USA). Each analyte was quantified using the ratios of each analyte peak area and the internal standard area. Deuterium-labeled compounds of target analytes were used as internal standards. The concentrations of the six analytes were further divided by the concentration of CR to correct a dilution factor. CR was analyzed simultaneously with six neurotransmitters by LC-MS. Differential analysis between high- and low-stress groups was performed using Excel (Microsoft; Redmond, WA, USA).

To generate the stress index, all analyte concentrations were transformed to logarithm. Then, the values were normalized by zero-mean-centering and unit-variance-scaling. Finally, the stress index was obtained by a linear combination of these values with the weight factors. The weights were optimized using the Excel-solver program, which minimizes *p*-values of Student’s *t*-tests between two groups.

### Method validation

We assessed the precision, accuracy, and robustness of the newly developed method as follows. The linearity of the calibration curves was assessed in the range of 0.01 to 1 μg/mL (5-HIAA, DA, GABA, and 5-HT) and 1 to 100 μg/mL (Cre, HVA, and VMA). The linear regression coefficients and relative errors (RE) from the predicted values of each concentration were evaluated. Within-run (intra) and between-run (inter) precision and accuracy were assessed using both authentic standards and human urine at five separate times on three separate days. The stability of six analytes in urine was assessed at room temperature for 24 h, at 4°C for 24 h, 3 days, and 7 days: and at -20°C for 24 h, 3 days, and 7 days. Post-preparation stability in solution A at 4°C for 48 h was investigated using both authentic standards and human urine.

## Results

### Method validation

The chromatograms and standard curves of six urinary neurotransmitters are shown in S1 Fig. The acceptable tolerance of the RE% and coefficient of variation (CV%) were estimated as 15% according to the Bioanalytical Method Validation Guidance for Industry provided by the Food and Drug Administration. All the regression coefficients of the six standard curves exceeded 0.99, and the RE from the predicted values to the observed concentrations were less than 15% within the range of 0.01–1 μg/mL for 5-HIAA, DA, GABA, and 5-HT, and 1–100 μg/mL for HVA and VMA (S2 Table). Within-run accuracies (RE%) and precisions (CV%) using authentic standards were both less than 15%, while only the accuracy of HVA slightly exceeded the tolerance (17%, S3 Table). Between-run (inter) accuracies (RE%) and precisions (CV%) using authentic standards were both less than 15% (S3 Table). Within- and between-run precisions (CV%) using urine were less than 15%; however, only within-run precision of HVA exceeded the tolerance (19.4%, S4 Table). The stability in urine was assessed at room temperature for 24h, at 4°C for 24h, 3 days, and 7 days; and at -20°C for 24 h, 3 days, and 7 days. As a result, it was revealed that all analytes were stable (< 15%) at room temperature within 24 h, or at -20°C within 7 days. Although the stability of VMA at -20°C for 3 days slightly exceeded 15%, we judged that it may attributed to analytical errors because it was within the tolerance over 7 days (S5 Table). HVA was not stable at 4°C (> 15%) even within 24 h, and VMA was not stable at 4°C (> 15%) over 7 days.

The post-preparation stability (stability on LC autosampler) of standards and urine extracts were both stable (< 15%) at 4°C for 48 h (S6 Table). We concluded that the accuracy, precision, and stability of this method were acceptable; however, the interpretation of HVA requires careful consideration because of larger errors.

### The relationship between BJSQ and CES-D

**Fig 2:**
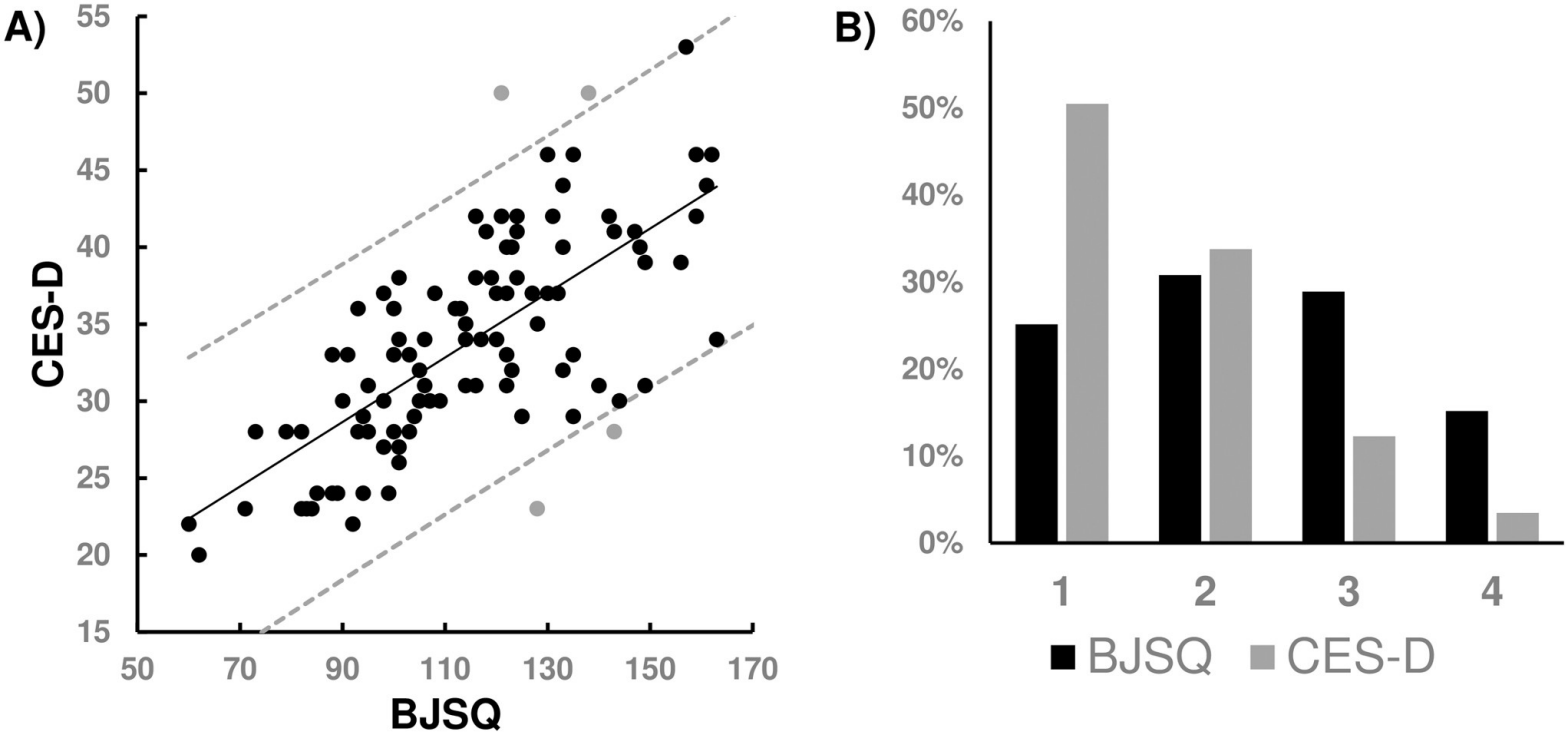
Comparison between BJSQ and CES-D. A scatter plot of BJSQ and CES-D total scores is shown in A) with a fitting curve and 95% CI curves. The ratio of four scores, 1 (low stress) to 4 (high stress) are plotted with bar graph B); black (BJSQ), gray (CES-D).

The vulnerability of this study is that it relied on a self-report questionnaire to define people with some mental stresses. Some participants may not answer the items honestly or may not understand their health conditions well by themselves. To check the reliability of the BJSQ answers, we compared the total BJSQ scores with the total CES-D scores using a scatter plot (Fig 2A). Since some items are common between two questionnaires, if the participants answered the items accurately, there would be a positive correlation. Otherwise, outliers would be observed. As a result, the coefficient of correlation reached 0.69, and abnormal outliers were not observed. Therefore, we determined that the answers of BJSQ were adequately reliable, and the BJSQ responses reflect participants’ mental state conditions accurately.

Fig 2B shows the distributions of the BJSQ and CES-D responses. It is obvious that the CES-D responses were highly biased to “1,” and the ratio of “4” was small, whereas the ratio of the four responses on the BJSQ were almost equal. Since the CES-D queries about major depressive disorder, healthy people rarely choose “3” and “4” if they feel stressed. In contrast, since the BJSQ measures job stress, participants choose “3” or “4” more easily.

### The relationship between neurotransmitter levels and mental health conditions

To clarify the relationship between the urinary neurotransmitters and stress factors, we first assigned participants into two groups for every item. The high-stress group included participants who chose 3 or 4 and the low-stress group included those who answered 1 or 2. Student’s *t*-tests between the two groups were performed for six neurotransmitters for all 57 items. The markers whose *p*-values were less than 0.01 are listed in Table 1 with the items. Urinary DA responded to participants’ dissatisfaction; e.g., “This job does not suit me well” and “I have felt gloomy.” It was also related to physical fatigue and pain; e.g., “I have felt extremely tired” and “I have experienced joint pains”.

**Table 1:** Significant responses of neural transmitters.

Marker	Questions		High (3+4)			Low (1+2)			Average difference	
			Ave.	N	95%CI	Ave.	n	95%CI	Δ Ave	*p*-value
DA	Q16	This job **does not** suit me well	0.586	23	(0.528–0.644)	0.759	77	(0.689–0.829)	0.173	0.00023
	Q24	I have felt extremely tired	0.628	34	(0.578–0.678)	0.766	66	(0.685–0.847)	0.138	0.0045
	Q33	I have felt gloomy	0.597	14	(0.536–0.658)	0.739	86	(0.674–0.804)	0.142	0.0018
	Q37	I have experienced joint pains	0.607	18	(0.546–0.669)	0.744	82	(0.676–0.811)	0.137	0.0034
GABA	Q43	I have experienced stomach and / or intestine problems	0.223	10	(0.160–0.286)	0.353	90	(0.302–0.404)	0.130	0.0021
	Q44	I have lost my appetite	0.232	15	(0.169–0.295)	0.359	85	(0.306–0.412)	0.127	0.0028
HVA	Q15	My working environment is poor (e.g. noise, lighting, temperature, ventilation)	10.6	17	(9.17–12.1)	13.2	83	(12.0–14.4)	2.6	0.0073
	Q46	I haven’t been able to sleep well	10.8	18	(9.44–12.1)	13.2	82	(12.0–14.4)	2.4	0.0078
	Q52	My spouse, family, friends, etc. are **not** reliable when I am troubled	11.0	22	(9.81–12.1)	13.3	78	(12.0–14.6)	2.3	0.007
5-HT	Q28	I have felt worried or insecure	0.220	16	(0.194–0.246)	0.268	84	(0.243–0.293)	0.048	0.0084
	Q37	I have experienced joint pains	0.204	18	(0.185–0.222)	0.273	82	(0.248–0.298)	0.069	0.000021
	Q43	I have experienced stomach and / or intestine problems	0.216	10	(0.190–0.242)	0.265	90	(0.242–0.289)	0.049	0.0055
VMA	Q2	I can’t complete work in the required time	6.24	25	(5.70–6.79)	7.63	75	(6.76–8.50)	1.39	0.0078

A significant decrease in urinary GABA was observed in people who experienced stomach and/or intestinal problems, or in those who had lost their appetite. Since GABA is related to glucagon secretion [38], its urinary level may be influenced by stomach and intestinal problems, or low appetite. HVA responded to poor working environments (“My working environment is poor”), sleep disturbance (“I haven’t been able to sleep well”), and isolation (“My spouse, family, friends, etc. are not reliable when I am troubled.”). VMA was related to uncontrollable situation (“I can’t complete work in the required time”). A significant decrease in urinary 5-HT was also observed in participants who felt worried or insecure, experienced joint pains, or stomach and intestine problems.

### Response of neurotransmitters to total BJSQ scores

**Fig 3:**
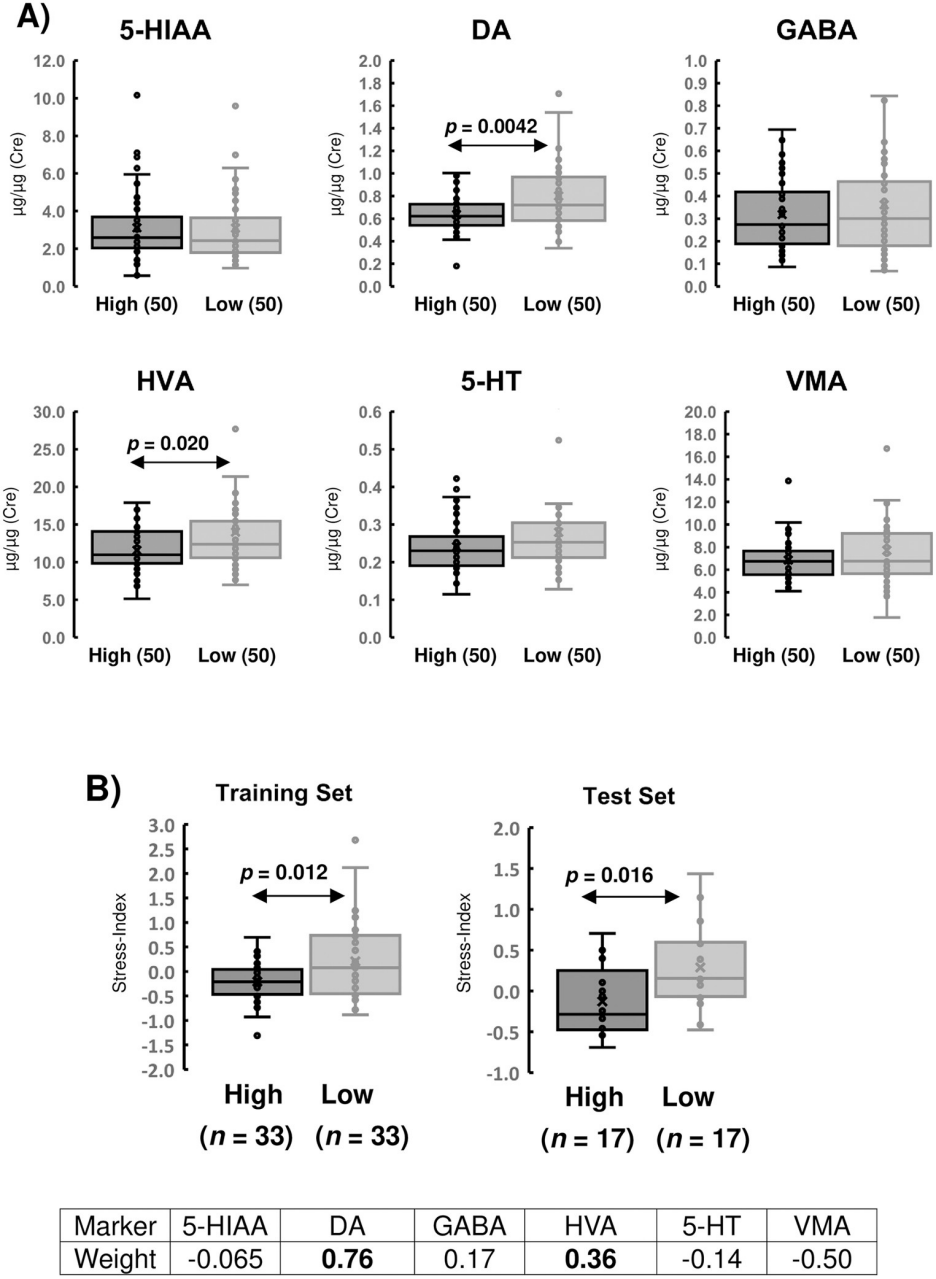
The expressions of six urinary neurotransmitters and their combined index. The examinees were divided into two groups based on their total BJSQ scores. The box-whisker plots of 5-HIAA, DA, GABA, HVA, 5-HT, and VMA are shown in A). Student’s *t*-tests were performed for the six urinary neurotransmitters, and *p*-values below 0.05 are shown in the plots. To generate the stress index, participants were randomly divided into a training (66) or test (34) set, and all analyte concentrations were logarithmically converted and normalized by zero-mean-centering and unit-variance-scaling. The stress index was generated by a linear combination of six normalized values with weight factors. The weight factors were optimized using the Excel-solver program B).

To evaluate the total stress severity, we compared the concentrations of six urinary transmitters with the total BJSQ scores. The 100 participants were divided into two groups according to their total BJSQ scores. The cut-off was determined by the statists provided by Ministry of Health, Labour and Welfare (MHLW) in Japan [39]. Before summing up the 57 BJSQ scores, the scores for the positive items; e.g., “I felt hopeful about the future” were reversed to match the stress severities. The high-stress group included 50 participants (27 men and 23 women; age = 49.6 ± 9.4 years), and the low-stress group included 50 volunteers (22 men and 28 women; age = 46.8 ± 12.0 years). When Student *t*-tests were performed for 36 laboratory test items between the two groups, there were no significant differences (*p* > 0.05), which means there were no biases between the two groups except for mental stress (Table 2). When comparing the concentrations of the six urinary neurotransmitters between the two groups, we observed a significant decrease in DA levels in the high-stress group (0.637 μg/mL) compared to the low-stress group (0.801 μg/mL, p = 0.0042). Similarly, HVA, which is the end-point metabolite of DA, showed a decrease in the high-stress group (11.6 μg/mL) compared to the low-stress group (14.0 μg/mL, p = 0.020, Fig 3A and Table 3). This suggests that urinary DA and its end-point metabolite HVA could be promising markers to distinguish total mental stress.

**Table 2:** Demographic features.

Item	High stress (*n* = 50)		Low stress (*n* = 50)		*p*-value
	Ave	95% CI	Ave	95% CI	
Gender	Male = 27, Female = 23		Male = 22, Female = 28		-
Age	49.6	(47.0–52.3)	46.8	(43.4–50.2)	0.193
BMI	22.6	(21.9–23.3)	22.1	(21.4–22.7)	0.247
TP	7.20	(7.11–7.30)	7.33	(7.24–7.42)	0.053
ALP	187	(172–201)	176	(159–193)	0.352
AST	19.2	(17.7–20.7)	19.3	(18.1–20.5)	0.900
ALT	17.1	(14.6–19.6)	16.5	(14.0–18.9)	0.714
LDH	166	(159–172)	161	(155–167)	0.295
γ-GTP	26.3	(21.6–30.9)	24.6	(19.7–29.5)	0.616
T-BIL	0.768	(0.709–0.827)	0.818	(0.732–0.904)	0.338
CPK	113	(98.8–127)	104	(90.9–118)	0.380
T-Ch	197	(190–204)	192	(183–201)	0.374
LDL-Ch	113	(107–119)	107	(99.8–114)	0.208
HDL-Ch	62.8	(59.6–66.0)	64.5	(61.0–67.9)	0.473
TG	84.0	(71.6–96.3)	73.3	(64.3–82.3)	0.163
NA	140	(140–141)	141	(140–141)	0.550
K	4.39	(4.28–4.49)	4.28	(4.18–4.38)	0.125
CL	104	(103–104)	104	(103–104)	0.854
CA	9.11	(9.02–9.20)	9.17	(9.09–9.24)	0.303
Mg	2.32	(2.27–2.37)	2.31	(2.25–2.37)	0.761
BUN	12.8	(12.0–13.7)	12.2	(11.2–13.1)	0.305
Cre	0.769	(0.726–0.812)	0.735	(0.692–0.777)	0.255
UA	5.20	(4.84–5.56)	5.02	(4.72–5.33)	0.450
BS	90.2	(87.6–92.9)	87.4	(85.0–89.7)	0.112
Alb	4.42	(4.36–4.48)	4.47	(4.41–4.53)	0.204
HbA1c	5.38	(5.31–5.46)	5.32	(5.26–5.39)	0.250
WBC	5.14	(4.75–5.54)	4.90	(4.57–5.23)	0.354
RBC	463	(451–475)	459	(447–471)	0.674
Hb	14.1	(13.7–14.5)	13.9	(13.5–14.4)	0.657
Ht	42.3	(41.2–43.4)	41.8	(40.6–42.9)	0.505
MCV	91.5	(90.1–93.0)	91.0	(89.4–92.6)	0.640
MCH	30.4	(29.9–31.0)	30.4	(29.8–30.9)	0.868
MCHC	33.3	(32.9–33.7)	33.4	(33.1–33.7)	0.678
NEUT	54.3	(51.8–56.7)	54.5	(52.1–56.9)	0.890
EOS	3.63	(2.52–4.73)	2.78	(2.11–3.45)	0.191
BAS	0.704	(0.583–0.825)	0.692	(0.604–0.780)	0.873
MON	5.77	(5.43–6.11)	5.55	(5.19–5.91)	0.387
LYM	35.7	(33.4–37.9)	36.5	(34.2–38.8)	0.602
PLT	26.4	(25.0–27.8)	26.3	(24.7–27.9)	0.936

**Table 3:** Average concentrations of urinary neurotransmitters in high and low stress groups.

		5-HIAA	DA	GABA	HVA	5-HT	VMA
High stress	Ave.	3.12	0.637	0.318	11.6	0.241	6.85
	St. dev.	0.263	0.0225	0.0215	0.407	0.00959	0.248
	95% CI Upper	3.64	0.682	0.361	12.4	0.261	7.35
	95% CI Lower	2.59	0.592	0.275	10.8	0.222	6.35
Low stress	Ave.	3.09	0.801	0.361	14.0	0.280	7.71
	St. dev.	0.277	0.0506	0.0422	0.923	0.0190	0.627
	95% CI Upper	3.65	0.903	0.446	15.8	0.318	8.97
	95% CI Lower	2.54	0.700	0.277	12.1	0.241	6.46
Δ Ave.		-0.0215	0.164	0.0430	2.41	0.0383	0.864
*p*		0.955	0.00420	0.366	0.0198	0.0763	0.204

When we divided the 100 patients into two groups according to CES-D total scores, we observed slight decreases in DA and HVA; however, their degrees were limited (*p* > 0.05). We concluded that the CES-D has a limit in recognizing mild mental stresses and is inappropriate for the classification of healthy persons’ mental conditions.

### An indicator reflecting total mental stress

Scatter plots of the two analyte concentrations for all combinations of six neurotransmitters are shown in S2 Fig. Some combinations showed relatively high correlations, such as 5-HIAA and GABA (*r* = 0.70), DA and 5-HT (*r* = 0.73), HVA and VMA (*r* = 0.75), and 5-HT and VMA (*r* = 0.72). However, most combinations showed poor correlations (*r* < 0.70), which means that the expression patterns of six urinary neurotransmitters are independent and the relationships are complementary. Therefore, the combination of six analytes is expected to increase the accuracy of mental stress identification.

To provide a simple and useful indicator that reflects total stress severities, we generated a combination index (stress index) from six neurotransmitters. First, 100 participants were randomly divided into two sets: training (66) and test (34). Both sets included an equal number of high- and low-stress persons. To generate the stress index, all analyte concentrations were logarithmically converted, and the values were normalized by zero-mean-centering and unit-variance-scaling. The stress index was generated by a linear combination of six normalized values with weight factors. The weight factors were optimized using the Excel-solver program, which minimizes *p*-values of Student’s *t*-tests between high- and low-stress groups in a training set. The stress index of the test set was calculated using the weights obtained from the training set and Student’s *t*-tests between high and low groups in the test set was calculated. As a result, the weights of DA (0.76) and HVA (0.36) were much higher than those of the others (5-HT: -0.14, VMA: -0.50, 5-HIAA: -0.065, and GABA: 0.17), which supports the result of a significant decrease in DA and HVA in the high-stress group (Fig 3A). The index values of the training set exhibited a significant difference between the high and low stress groups, with values of -0.21 and 0.21 respectively (*p* = 0.012). Similarly, in the test set, the index values showed a significant change from -0.13 (high) to 0.29 (low) (*p* = 0.016). These findings highlight the potential of the stress index, generated from six urinary neurotransmitters, as a promising indicator for identifying overall mental stress levels (Fig 3B).

## Discussion

Employees in Japan were historically encouraged to work long hours, leading to a rise in mental pressure and overwork-related deaths during the 2000s. In response to the increasing number of suicides associated with overwork, the Japanese government implemented the BJSQ in all companies with more than 50 employees. While this measure improved working environments and their management, depression resulting from poor work environments still persists as a social problem.

We focused on utilizing urinary neurotransmitters as potential biomarkers for mental stress assessment. Non-invasive urine sampling offers advantages in terms of convenience and ease of collection, especially when identifying mental stress in healthy individuals outside of clinical settings. A key challenge in our study was accurately identifying and recruiting individuals with asymptomatic mental stress. To address this challenge, we employed the BJSQ, which assesses not only mental stress but also work environments and personal lives, providing a comprehensive evaluation of stress levels suitable for our study’s objectives.

We evaluated the degree of mental stress by analyzing the levels of six neurotransmitters (DA, 5-HT, 5-HIAA, GABA, HVA, and VMA) in urine samples obtained from healthy volunteers. We observed that the concentration of each urinary neurotransmitter varied depending on different stress factors. For example, DA responded to dissatisfied moods or physical fatigue, GABA was related to appetite, HVA responded to working environments, sleep disturbances, and isolation, VMA was associated with uncontrollable situations, and 5-HT was linked to anxiety. Both DA and HVA also responded to total stress severities, indicating that the "stress index" generated from the six urinary biomarkers could be a promising indicator to determine overall stress severities. Considering that the study consists of only healthy volunteers, not including patients with depression, these urinary neurotransmitters show promise as biomarkers for detecting mental stress in healthy individuals.

Furthermore, as mentioned in the introduction, recent research has examined the association between changes in urinary neurotransmitters and various disorders. For example, a study by Kheirandish-Gozal et al. on children with pediatric obstructive sleep apnea found that overnight increases in urinary epinephrine, norepinephrine, and GABA levels were linked to cognitive dysfunction. They hypothesized that elevated GABA levels might contribute to mechanisms of neuronal excitotoxicity and dysfunction in this population [40]. Additionally, Peacock et al. conducted a retrospective analysis comparing urinary catecholamines and neurotransmitters in depression patients to healthy controls. They discovered significant alterations in these biomarkers, suggesting potential interactions among psychological stress, inflammation, and oxidative stress pathways in depression [41].

Several limitations of this study should be acknowledged. Firstly, the influence of sampling timing on urinary neurotransmitter levels needs to be assessed. Previous research has shown diurnal variations in urinary HVA and VMA levels, with healthy individuals exhibiting a gradual decrease throughout the day, while patients with depression maintain low levels throughout the day. As all samplings were conducted in the morning in this study, observed differences might be diminished if samplings were randomly performed at different times. Therefore, future studies should carefully design the sampling period and timing to establish a robust screening test. Secondly, due to the limited impact of mental stress on urinary neurotransmitter changes, a larger sample size may yield more definitive outcomes. Furthermore, omics approaches, such as proteomics and metabolomics, may contribute to find new urinary biomarkers that respond to mental stress [42–44]. Thirdly, the effectiveness of early interventions, such as improving the working environment or removing stress factors, needs to be assessed based on empirical evidence. Lastly, while neurotransmitters are primarily synthesized in the brain, they are also produced in other organs such as the kidneys or gut. Additionally, most neurotransmitters cannot pass through the blood-brain barrier, suggesting that the roles of neurotransmitters in the brain and other organs are independent. However, several studies have demonstrated that serum or urinary neurotransmitter levels can reflect mental states, including depression and schizophrenia. Therefore, it is crucial to investigate and clarify the underlying reasons why urinary neurotransmitters can serve as indicators of an individual’s mental state. By acknowledging these limitations and addressing them in future research, we can further advance our understanding of the relationship between urinary neurotransmitters and stress levels and develop more reliable and applicable screening tests.

## Conclusions

We developed a simultaneous analysis of urinary neurotransmitters using mass spectrometry. We revealed that the concentration of these urinary neurotransmitters changed depending on various stress factors, and the “stress index” generated from six urinary biomarkers was a promising indicator to determine the total stress severities. This may provide clues for scientists to develop new screening tests for mental stress or depression. Further research and validation are required to establish the utility and accuracy of urinary neurotransmitter analysis as a diagnostic tool for mental stress.

## Supporting information

S1 Text The Brief Job Stress Questionnaire (BJSQ). (DOCX)

S2 Text Center for Epidemiologic Studies Depression Scale (CES-D). (DOCX)

S1 Table MRM transitions. (DOCX)

S2 Table Method Validation of LC-MS, linearity of standard curves. (DOCX)

S3 Table Method Validation of LC-MS, Within-run (intra) and between-run (inter) precision and accuracy using authentic standards. (DOCX)

S4 Table Method Validation of LC-MS, Within-run (intra) and between-run (inter) precision and accuracy using human urine. (DOCX)

S5 Table Method Validation, stability in urine. (DOCX)

S6 Table Method Validation, stability at 4°C after preparation for 48 hours. (DOCX)

S1 Fig LC-MS/MS chromatograms and standard curves of six neurotransmitters. (TIF)

S2 Fig Correlation between two neurotransmitters’ expressions. (TIF)

S1 File Laboratory tests. (ZIP)

S2 File Concentrations of six analytes and BJSQ and CES-D scores. (ZIP)

S1 Checklist STROBE statement—Checklist of items that should be included in reports of observational studies. (PDF)

## Abbreviations

5-HIAA: 5-hydoroxyindoleacetic acid
5-HT: 5-hydoroxytoryptamine serotonin
BJSQ: Brief Job Stress Questionnaire
CES-D: The Center for Epidemiologic Studies Depression Scale
Cre: creatinine
CV: coefficient of variation
DA: dopamine
GABA: gamma-aminobutyric acid
HVA: homovanillic acid
LC-MS: liquid chromatography-mass spectrometer
QC: quality control
RE: relative error
VMA: vanillylmandelic acid
