# Supplementary material for: Mental stress objective screening for workers using urinary neurotransmitters
Source: PLoS One. 2023 Sep 8;18(9):e0287613. doi: 10.1371/journal.pone.0287613 (PMC10490881; doi:10.1371/journal.pone.0287613)
Supplement: S1 Text — (DOCX) [file pone.0287613.s001.docx]

**S1 Text**: The Brief Job Stress Questionnaire (BJSQ)

1. Please answer the following questions concerning your job by circling the number that best fits your situation.

A. Very much so, B. Moderately so, C. Somewhat, D. Not at all

1) I have an extremely large amount of work to do

2) I cannot complete work in the required time

3) I have to work as hard as I can

4) I have to pay very careful attention

5) My job is difficult in that it requires a high level of knowledge and technical skill

6) I need to be constantly thinking about work throughout the working day

7) My job requires a lot of physical work

8) I can work at my own pace

9) I can choose how and in what order to do my work

10) I can reflect my opinions on workplace policy

11) My knowledge and skills are rarely used at work

12) There are differences of opinion within my department

13) My department does not get along well with other departments

14) The atmosphere in my workplace is friendly

15) My working environment is poor (e.g. noise, lighting, temperature, ventilation)

16) This job suits me well

17) My job is worth doing

2. Please answer the following questions concerning your health during the past month by circling the number that best fits your situation.

A. Almost never, B. Sometimes, C. Often, D. Almost always

18) I have been very active

19) I have been full of energy

20) I have been lively

21) I have felt angry

22) I have been inwardly annoyed or aggravated

23) I have felt irritable

24) I have felt extremely tired

25) I have felt exhausted

26) I have felt weary or listless

27) I have felt tense

28) I have felt worried or insecure

29) I have felt restless

30) I have been depressed

31) I have thought that doing anything was a hassle

32) I have been unable to concentrate

33) I have felt gloomy

34) I have been unable to handle work

35) I have felt sad

36) I have felt dizzy

37) I have experienced joint pains

38) I have experienced headaches

39) I have had a stiff neck and / or shoulders

40) I have had lower back pain

41) I have had eyestrain

42) I have experienced heart palpitations or shortness of breath

43) I have experienced stomach and / or intestine problems

44) I have lost my appetite

45) I have experienced diarrhea and / or constipation

46) I haven’t been able to sleep well

3. Please answer the following questions concerning satisfaction by circling the number that best fits your situation.

A. Extremely, B. Very much, C. Somewhat, D. Not at all

3.1. How freely can you talk with the following people?

47) Superiors

48) Co-workers

49) Spouse, family, friends, etc.

3.2. How reliable are the following people when you are troubled?

50) Superiors

51) Co-workers

52) Spouse, family, friends, etc.

3.3. How well will the following people listen to you when you ask for advice on personal matters?

53) Superiors

54) Co-workers

55) Spouse, family, friends, etc.

4. Please answer the following questions concerning satisfaction by circling the number that best fits your situation.

A. Satisfied, B. Somewhat satisfied, C. Somewhat dissatisfied, D. Dissatisfied

56) I am satisfied with my job

57) I am satisfied with my family life
