# Supplementary material for: Mental stress objective screening for workers using urinary neurotransmitters
Source: PLoS One. 2023 Sep 8;18(9):e0287613. doi: 10.1371/journal.pone.0287613 (PMC10490881; doi:10.1371/journal.pone.0287613)
Supplement: S1 Table — (DOCX) [file pone.0287613.s003.docx]

**S1 Table:** MRM Transitions

|  | Precursor | Product | CE (V) | Polarity | Q1  resolution | Q2  resolution |
| --- | --- | --- | --- | --- | --- | --- |
| 5-HIAA | 192.1 | 146.0 | 16 | Positive | Unit | Unit |
| DA | 154.1 | 91.0 | 24 | Positive | Unit | Unit |
| GABA | 104.1 | 87.0 | 8 | Positive | Unit | Unit |
| HVA | 181.1 | 137.0 | 4 | Negative | Unit | Unit |
| 5-HT | 177.1 | 160.1 | 24 | Positive | Unit | Unit |
| VMA | 197.0 | 137.0 | 24 | Negative | Unit | Unit |
| Cre | 114.1 | 44.2 | 24 | Positive | Unit | Unit |
| DA-d4 | 158.1 | 141.1 | 8 | Positive | Unit | Unit |
| GABA-d6 | 110.1 | 93.0 | 8 | Positive | Unit | Unit |
| 5-HT-d4 | 181.1 | 164.0 | 8 | Positive | Unit | Unit |
| HVA-d3 | 184.1 | 140.0 | 4 | Negative | Unit | Unit |
| VMA-d3 | 200.1 | 136.9 | 20 | Negative | Unit | Unit |
| Cre-d3 | 117.1 | 47.1 | 16 | Positive | Unit | Unit |
