# Supplementary material for: Mental stress objective screening for workers using urinary neurotransmitters
Source: PLoS One. 2023 Sep 8;18(9):e0287613. doi: 10.1371/journal.pone.0287613 (PMC10490881; doi:10.1371/journal.pone.0287613)
Supplement: S2 Table — (DOCX) [file pone.0287613.s004.docx]

**S2 Table**: Method Validation of LC-MS

Table S4-1: Linearity of Standard Curves

| (µg/mL) | | S1 | S2 | S3 | S4 | S5 | R^2^ |
| --- | --- | --- | --- | --- | --- | --- | --- |
| 5-HIAA | Set Value | 0.01 | 0.03 | 0.1 | 0.3 | 1 | 0.997 |
|  | Quantified | 0.0102 | 0.0288 | 0.0941 | 0.316 | 1.03 |  |
|  | RE (%) | 1.7 | -4.0 | -5.9 | 5.4 | 2.8 |  |
| DA | Set Value | 0.01 | 0.03 | 0.1 | 0.3 | 1 | 0.991 |
|  | Quantified | 0.0104 | 0.0256 | 0.105 | 0.299 | 1.05 |  |
|  | RE (%) | 4.3 | -14.8 | 5.3 | -0.2 | 5.2 |  |
| GABA | Set Value | 0.01 | 0.03 | 0.1 | 0.3 | 1 | 0.998 |
|  | Quantified | 0.0102 | 0.0284 | 0.103 | 0.294 | 1.03 |  |
|  | RE (%) | 1.5 | -5.2 | 2.6 | -1.9 | 3.0 |  |
| 5-HT | Set Value | 0.01 | 0.03 | 0.1 | 0.3 | 1 | 0.999 |
|  | Quantified | 0.0101 | 0.0286 | 0.102 | 0.293 | 1.03 |  |
|  | RE (%) | 1.3 | -4.5 | 2.5 | -2.3 | 3.1 |  |
| Cre | Set Value | 1 | 3 | 10 | 30 | 100 | 0.993 |
|  | Quantified | 1.01 | 2.78 | 11.2 | 28.8 | 97.9 |  |
|  | RE (%) | 1.4 | -7.2 | 11.9 | -3.9 | -2.1 |  |
| HVA | Set Value | 1 | 3 | 10 | 30 | 100 | 0.996 |
|  | Quantified | 0.99 | 3.09 | 10.3 | 31.3 | 91.2 |  |
|  | RE (%) | -1.3 | 3.0 | 2.7 | 4.5 | -8.8 |  |
| VMA | Set Value | 1 | 3 | 10 | 30 | 100 | 0.998 |
|  | Quantified | 1.01 | 2.94 | 9.87 | 28.9 | 106 |  |
|  | RE (%) | 0.9 | -2.1 | -1.3 | -3.8 | 6.4 |  |
