# Supplementary material for: Mental stress objective screening for workers using urinary neurotransmitters
Source: PLoS One. 2023 Sep 8;18(9):e0287613. doi: 10.1371/journal.pone.0287613 (PMC10490881; doi:10.1371/journal.pone.0287613)
Supplement: S3 Table — (DOCX) [file pone.0287613.s005.docx]

**S3 Table**: Within-run (intra) and between-run (inter) precision and accuracy using authentic standards

|  | | Within-run (Intra) *n* = 5 | | Between-run (Inter) *n* = 5 × 3 days | |
| --- | --- | --- | --- | --- | --- |
| Markers | (µg/mL) | RE | CV | RE | CV |
|  |  | (%) | (%) | (%) | (%) |
| 5-HIAA | 0.05 | -7.0 | 1.4 | -5.8 | 9.2 |
| DA | 0.05 | -3.0 | 1.2 | -1.2 | 2.2 |
| GABA | 0.05 | 0.1 | 1.9 | 0.0 | 1.4 |
| 5-HT | 0.05 | -1.8 | 0.8 | -0.2 | 1.4 |
| Cre | 5 | 0.2 | 0.3 | 1.8 | 1.5 |
| HVA | 5 | 17.0 | 4.2 | 5.2 | 9.8 |
| VMA | 5 | 3.3 | 1.3 | 4.0 | 0.8 |
