# Supplementary material for: Mental stress objective screening for workers using urinary neurotransmitters
Source: PLoS One. 2023 Sep 8;18(9):e0287613. doi: 10.1371/journal.pone.0287613 (PMC10490881; doi:10.1371/journal.pone.0287613)
Supplement: S4 Table — (DOCX) [file pone.0287613.s006.docx]

**S4 Table**: Within-run (intra) and between-run (inter) precision and accuracy using human urine

|  | Within-run (Intra) *n* = 5 | | | Between-run (Inter) *n* = 5 × 3 days | | |
| --- | --- | --- | --- | --- | --- | --- |
| Biomarkers | AV  (µg/mL) | SD  (µg/mL) | CV | AV  (µg/mL) | SD  (µg/mL) | CV |
|  |  |  | (%) |  |  | (%) |
| 5-HIAA | 0.672 | 0.0116 | 1.7 | 0.646 | 0.0288 | 4.4 |
| DA | 0.486 | 0.0287 | 5.9 | 0.517 | 0.0377 | 7.3 |
| GABA | 0.124 | 0.00167 | 1.3 | 0.131 | 0.00651 | 14.2 |
| 5-HT | 0.124 | 0.00167 | 1.3 | 0.145 | 0.0187 | 4.2 |
| Cre | 687 | 45.9 | 6.7 | 689 | 5.29 | 0.8 |
| HVA | 7.38 | 1.43 | 19.4 | 7.24 | 0.140 | 2.0 |
| VMA | 2.95 | 0.273 | 9.2 | 3.19 | 0.245 | 7.7 |
