# Supplementary material for: Mental stress objective screening for workers using urinary neurotransmitters
Source: PLoS One. 2023 Sep 8;18(9):e0287613. doi: 10.1371/journal.pone.0287613 (PMC10490881; doi:10.1371/journal.pone.0287613)
Supplement: S5 Table — (DOCX) [file pone.0287613.s007.docx]

**S5 Table**: Stability in Urine

|  | RE (%) | | | | | | |
| --- | --- | --- | --- | --- | --- | --- | --- |
| Biomarkers | Room Temp. | 4 ℃ | | | -20 ℃ | | |
|  | 24 hours | 24 hours | 3 days | 7 days | 24 hours | 3 days | 7 days |
| 5-HIAA | 11.6 | 12.9 | -2.6 | -0.2 | 5.2 | 0.7 | -2.0 |
| DA | -7.5 | -2.4 | -0.4 | -4.1 | -2.7 | 1.4 | -6.5 |
| GABA | 5.4 | 6.7 | 1.1 | 2.5 | 3.4 | -0.1 | 0.8 |
| 5-HT | 1.0 | 8.5 | 1.0 | -1.2 | 1.1 | 2.8 | -9.1 |
| Cre | -0.4 | 4.9 | -2.5 | -0.3 | 2.6 | 4.2 | 0.9 |
| HVA | 4.9 | 19.0 | 30.2 | 21.3 | -2.9 | 6.0 | 12.9 |
| VMA | 12.3 | 13.0 | -8.9 | 22.3 | 0.8 | -15.5 | 1.0 |
