# Supplementary material for: Mental stress objective screening for workers using urinary neurotransmitters
Source: PLoS One. 2023 Sep 8;18(9):e0287613. doi: 10.1371/journal.pone.0287613 (PMC10490881; doi:10.1371/journal.pone.0287613)
Supplement: S6 Table — (DOCX) [file pone.0287613.s008.docx]

**S6 Table**: Post-preparation stability at 4 ℃ (on LC autosampler) for 48 hours

|  | Authentic Standards | | Urine | |
| --- | --- | --- | --- | --- |
| Biomarkers | Conc. | RE | Conc. | RE |
|  | (µg/mL) | (%) | (µg/mL) | (%) |
| 5-HIAA | 0.05 | -14.1 | 0.706 | 5.0 |
| DA | 0.05 | 14.6 | 0.522 | 7.5 |
| GABA | 0.05 | 8.5 | 0.134 | 7.3 |
| 5-HT | 0.05 | 4.9 | 0.152 | 5.0 |
| Cre | 5 | 3.0 | 690 | 0.4 |
| HVA | 5 | -4.7 | 7.19 | -2.6 |
| VMA | 5 | 10.1 | 3.25 | 10.1 |
