# Supplementary figures and images for: Mental stress objective screening for workers using urinary neurotransmitters
Source: PLoS One. 2023 Sep 8;18(9):e0287613. doi: 10.1371/journal.pone.0287613 (PMC10490881; doi:10.1371/journal.pone.0287613)

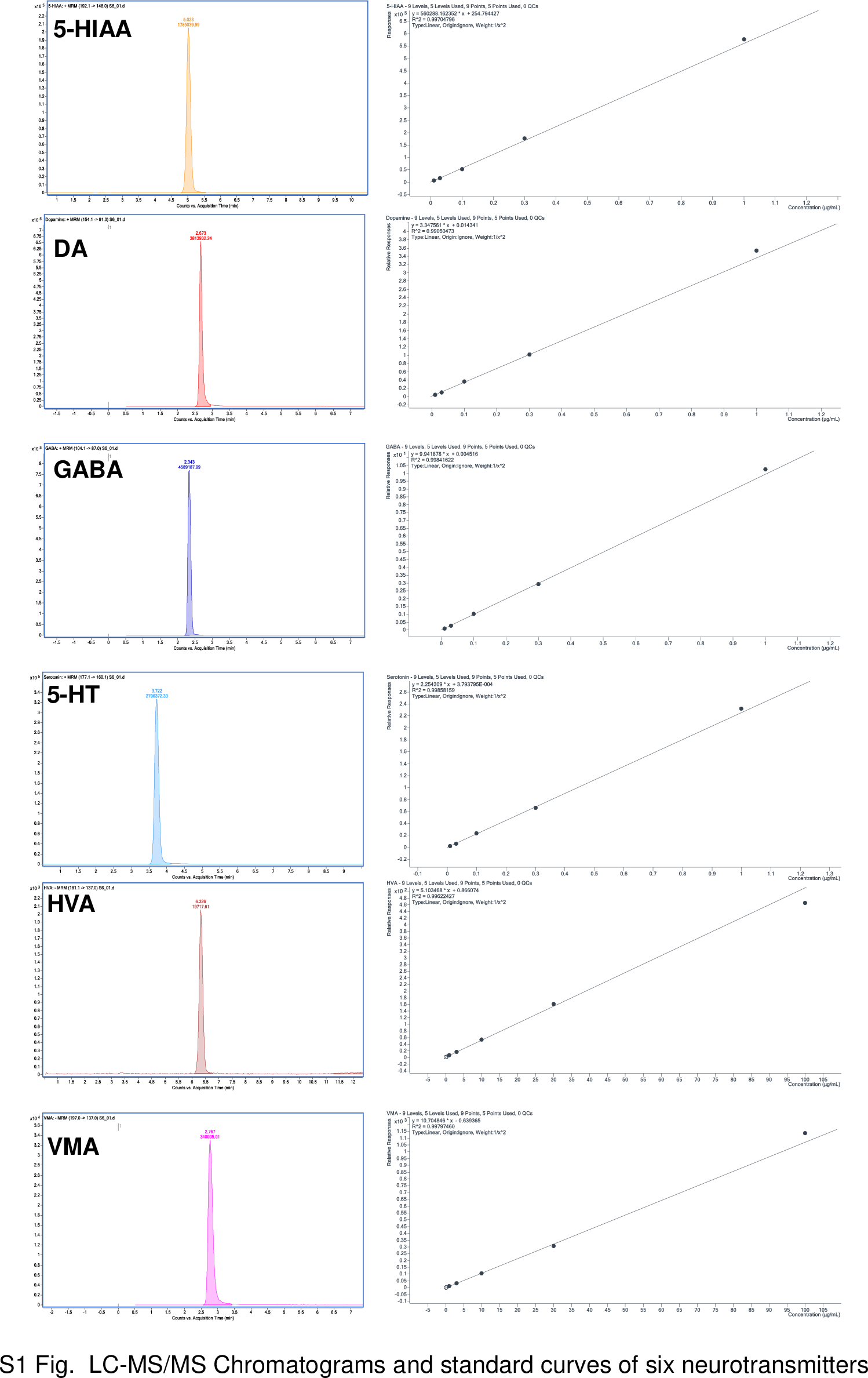

Supplement: S1 Fig — (TIF) [file pone.0287613.s009.tif]

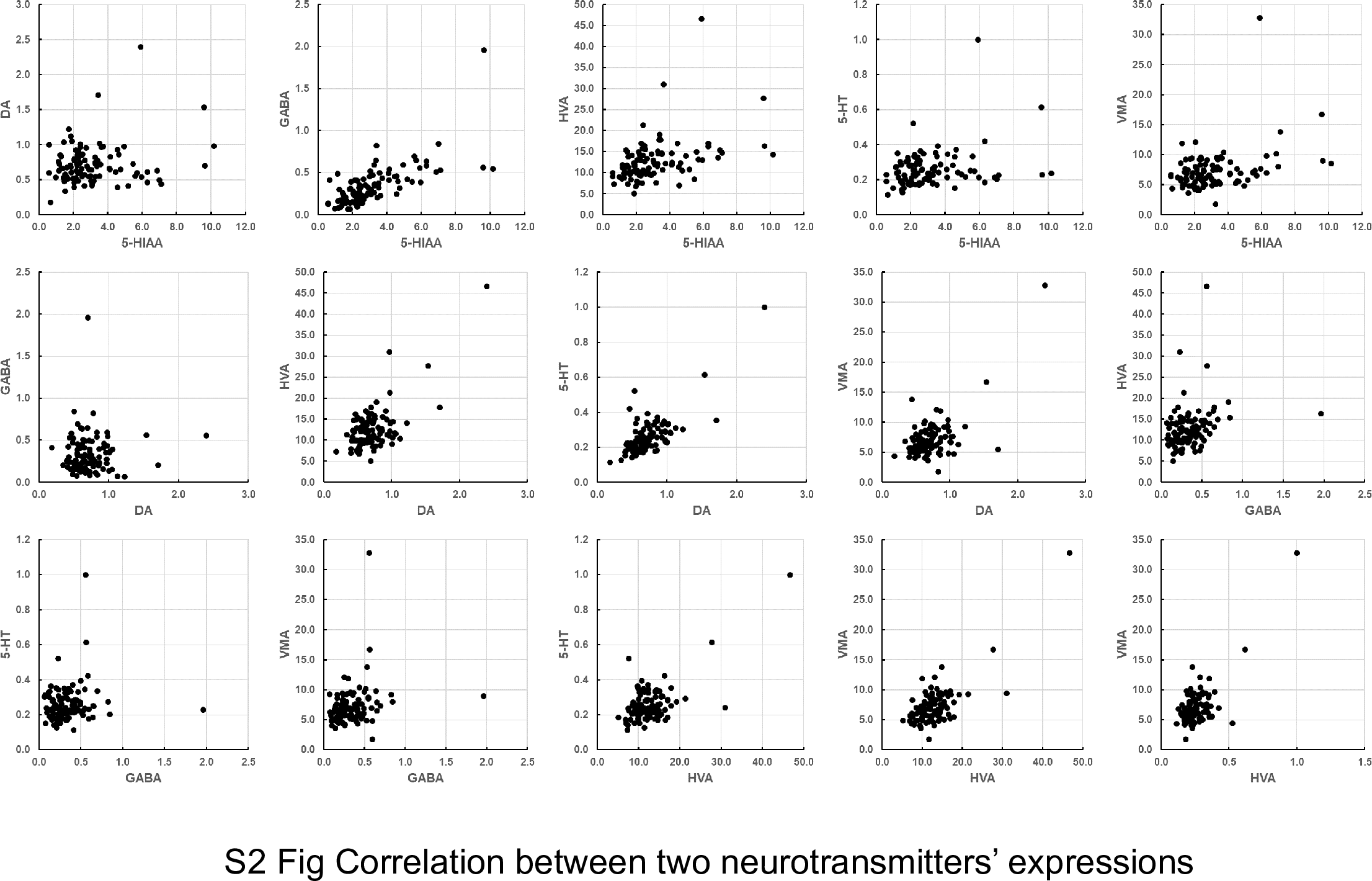

Supplement: S2 Fig — (TIF) [file pone.0287613.s010.tif]
