# Supplementary material for: Mental stress objective screening for workers using urinary neurotransmitters
Source: PLoS One. 2023 Sep 8;18(9):e0287613. doi: 10.1371/journal.pone.0287613 (PMC10490881; doi:10.1371/journal.pone.0287613)
Supplement: S1 Checklist — (PDF) [file pone.0287613.s013.pdf]

STROBE Statement—checklist of items that should be included in reports of observational studies

	Item No.	Recommendation	Page No.	Relevant text from manuscript
**Title and abstract**	1	(*a*) Indicate the study's design with a commonly used term in the title or the abstract	2	"We obtained urine specimens from 100 healthy volunteers after they received medical checks and answered the Brief Job Stress Questionnaire (BJSQ). Participants were divided into high- and low- stress groups according to their total BJSQ scores. We further analysed six urinary neurotransmitters using liquid chromatography-mass spectrometry."
		(*b*) Provide in the abstract an informative and balanced summary of what was done and what was found	2	"We obtained the concentrations of the six analytes from 100 examinees and revealed that the levels of urinary dopamine (p = 0.0042) and homovanillic acid (p = 0.020) were significantly lower in the high-stress group than those in the low-stress group."
**Introduction**				
Background/rationale	2	Explain the scientific background and rationale for the investigation being reported	4	"Approximately 2–15% of the population experiences depression or anxiety disorders at some point in their lives. However, diagnosing these disorders still relies on subjective assessments by doctors, making it challenging to identify pre-disease states with an increased risk of developing these disorders."
Objectives	3	State specific objectives, including any prespecified hypotheses	5	"We hypothesize that urinary neurotransmitter can still serve as potential biomarkers for monitoring mental health conditions and assessing the degree of mental stress. In this study, we aim to establish a robust and reliable screening test using urine samples from healthy volunteers, targeting six neurotransmitters (DA, 5-HT, 5-HIAA, GABA, HVA, and VMA)."
**Methods**				
Study design	4	Present key elements of study design early in the paper	6	"All participants started fasting from 9 p.m. and sampling and self-report questionnaires were conducted in the next morning. Serum, urine, and saliva and subjects' data were obtained from all participants during the above-mentioned period. All participants answered the BJSQ. The CES-D was also administered to all participants to check the accuracy of BJSQ answers. Participants were interviewed by medical doctors, which included items about smoking and alcohol drinking habits and their familial health histories. Finally, participants underwent laboratory tests including 36 items related to hepatic, renal, and metabolic functions."
Setting	5	Describe the setting, locations, and relevant dates, including periods of recruitment, exposure, follow-up, and data collection	6	"All participants were recruited by Soiken Inc. Participants were recruited from July 1 to 31, 2019 and written informed consents were obtained from all patients. All participants started

				fasting from 9 p.m. and sampling and self-report questionnaires were conducted in the next morning. “
Participants	6	(*a*) *Cohort study*—Give the eligibility criteria, and the sources and methods of selection of participants. Describe methods of follow-up<br>*Case-control study*—Give the eligibility criteria, and the sources and methods of case ascertainment and control selection. Give the rationale for the choice of cases and controls<br>*Cross-sectional study*—Give the eligibility criteria, and the sources and methods of selection of participants	-	No applicable
		(*b*) *Cohort study*—For matched studies, give matching criteria and number of exposed and unexposed<br>*Case-control study*—For matched studies, give matching criteria and the number of controls per case	-	No applicable
Variables	7	Clearly define all outcomes, exposures, predictors, potential confounders, and effect modifiers. Give diagnostic criteria, if applicable	24	“Described in Table2; Age, BMI, TP, ALP, AST, ALT, LDH, γ -GTP, T-BIL, CPK, T-Ch, LDL-Ch, HDL-Ch, TG, NA, K, CL, CA, Mg, BUN, Cre, UA, BS, Alb, HbA1c, WBC, RBC, Hb, Ht, MCV, MCH, MCHC, NEUT, EOS, BAS, MON, LYM, PLT”
Data sources/ measurement	8*	For each variable of interest, give sources of data and details of methods of assessment (measurement). Describe comparability of assessment methods if there is more than one group	9	“Some participants may not answer the items honestly or may not understand their health conditions well by themselves. To check the reliability of the BJSQ answers, we compared the total BJSQ scores with the total CES-D scores using a scatter plot (Fig 2A).”
Bias	9	Describe any efforts to address potential sources of bias	11	“When Student t-tests were performed for 36 laboratory test items between the two groups, there were no significant differences ($p > 0.05$), which means there were no biases between the two groups except for mental stress (Table 2).”

Study size	10	Explain how the study size was arrived at	6	“We assumed that the average concentration of neural transmitters would differ by no more than half of the standard deviations within each group. Based on this assumption, we estimated a sample size of approximately 50, considering an alpha-error (0.05) and beta-error (0.2) framework.”

Continued on next page

Quantitative variables	11	Explain how quantitative variables were handled in the analyses. If applicable, describe which groupings were chosen and why	6	"Participants chose one of four options depending on their mental conditions. Since both the BJSQ and CES-D include items that ask about both positive (e.g., "I felt hopeful about the future") and negative (e.g., "I felt depressed") feelings, we reversed the scores of the positive items before summing all scores."<br>For example, the participants chose one option from following options.<br>1. Please answer the following questions concerning your job by circling the number that best fits your situation.<br>A. Very much so, B. Moderately so, C. Somewhat, D. Not at all<br>"The 100 participants were divided into two groups according to their total BJSQ scores. The cut-off was determined by the statists provided by Ministry of Health, Labour and Welfare (MHLW) in Japan[39]."
Statistical methods	12	(*a*) Describe all statistical methods, including those used to control for confounding	-	In this study, we did not observe any confounding factors or biases, therefore, we did not do any correlations for the comparison.
		(*b*) Describe any methods used to examine subgroups and interactions	9	"To clarify the relationship between the urinary neurotransmitters and stress factors, we first assigned participants into two groups for every item. The high-stress group included participants who chose 3 or 4 and the low-stress group included those who answered 1 or 2. Student's t-tests between the two groups were performed for six neurotransmitters for all 57 items."
		(*c*) Explain how missing data were addressed	6	"We did not sum up scores of some items which the participants did not answer."
		(*d*) *Cohort study*—If applicable, explain how loss to follow-up was addressed<br>***Case-control study*—If applicable, explain how matching of cases and controls was addressed.**<br>*Cross-sectional study*—If applicable, describe analytical methods taking account of sampling strategy	11	"First, 100 participants were randomly divided into two sets: training (66) and test (34). Both sets included an equal number of high- and low-stress persons. To generate the stress index, all analyte concentrations were logarithmically converted, and the values were normalized by zero-mean-centering and unit-variance-scaling. The stress index was generated by a linear combination of six normalized values with weight factors. The weight factors were optimized using the Excel-solver program, which minimizes p-values of Student's t-tests between high- and low-stress groups in a training set. The stress index of the test set was calculated using the weights obtained from the training set and Student's t-tests between high and low groups in the test set was calculated.2
		(*e*) Describe any sensitivity analyses	9	"The vulnerability of this study is that it relied on a self-report questionnaire to define people with some mental stresses. Some participants may not answer the items honestly or may not understand their health conditions well by themselves. To check the reliability of the BJSQ

				answers, we compared the total BJSQ scores with the total CES-D scores using a scatter plot (Fig 2A).”
**Results**				
Participants	13*	(a) Report numbers of individuals at each stage of study—eg numbers potentially eligible, examined for eligibility, confirmed eligible, included in the study, completing follow-up, and analysed	-	In this study, we excluded participants with underlying health conditions, as determined by medical evaluation. However, the specific number of participants who were excluded at this stage was not recorded. Therefore, we are unable to provide exact figures for the numbers of individuals excluded based on health criteria.
		(b) Give reasons for non-participation at each stage	6	“Participants who were judged as unhealthy by the doctor (e.g., serious renal failure, hepatic failure, diabetes, etc.) were excluded.”
		(c) Consider use of a flow diagram	-	We did not show the flow diagram.
Descriptive data	14*	(a) Give characteristics of study participants (eg demographic, clinical, social) and information on exposures and potential confounders	6	“All participants were recruited by Soiken Inc. Participants were interviewed by medical doctors, which included items about smoking and alcohol drinking habits and their familial health histories. Participants who were judged as unhealthy by the doctor (e.g., serious renal failure, hepatic failure, diabetes, etc.) were excluded. “
		(b) Indicate number of participants with missing data for each variable of interest	6	“We did not sum up scores of some items which the participants did not answer.”
		(c) *Cohort study*—Summarise follow-up time (eg, average and total amount)	-	Not applicable
Outcome data	15*	*Cohort study*—Report numbers of outcome events or summary measures over time	-	- Not applicable
		Case-control study—Report numbers in each exposure category, or summary measures of exposure	10,11	“To clarify the relationship between the urinary neurotransmitters and stress factors, we first assigned participants into two groups for every item. The high-stress group included participants who chose 3 or 4 and the low-stress group included those who answered 1 or 2. Student’s t-tests between the two groups were performed for six neurotransmitters.”<br>“The cut-off was determined by the statists provided by Ministry of Health, Labour and Welfare (MHLW) in Japan[39]. Before summing up the 57 BJSQ scores, the scores for the positive items; e.g., “I felt hopeful about the future” were reversed to match the stress severities. The high-stress group included 50 participants (27 men and 23 women; age = 49.6 ± 9.4 years), and the low-stress group included 50 volunteers (22 men and 28 women; age = 46.8 ± 12.0 years).”

		Cross-sectional study—Report numbers of outcome events or summary measures	-	-
Main results	16	(*a*) Give unadjusted estimates and, if applicable, confounder-adjusted estimates and their precision (eg, 95% confidence interval). Make clear which confounders were adjusted for and why they were included	23,24	The 95% confidence intervals were shown in Table 1 and 2 for all variables, which were all unadjusted. The linear regression model was established with the training data set and the performance was evaluated with test data set.
		(*b*) Report category boundaries when continuous variables were categorized	11	"The 100 participants were divided into two groups according to their total BJSQ scores. The cut-off was determined by the statists provided by Ministry of Health, Labour and Welfare (MHLW) in Japan[39]."
		(*c*) If relevant, consider translating estimates of relative risk into absolute risk for a meaningful time period	-	We did not consider translating estimates of relative risk into absolute risk for a meaningful period.

Continued on next page

Other analyses	17	Report other analyses done—eg analyses of subgroups and interactions, and sensitivity analyses	11	"Scatter plots of the two analyte concentrations for all combinations of six neurotransmitters are shown in S2 Fig. Some combinations showed relatively high correlations, such as 5-HIAA and GABA (r = 0.70), DA and 5-HT (r = 0.73), HVA and VMA (r = 0.75), and 5-HT and VMA (r = 0.72). However, most combinations showed poor correlations (r < 0.70), which means that the expression patterns of six urinary neurotransmitters are independent, and the relationships are complementary. "
**Discussion**				
Key results	18	Summarise key results with reference to study objectives	13	"We observed that the concentration of each urinary neurotransmitter varied depending on different stress factors. DA responded to dissatisfied moods or physical fatigue, GABA was related to appetite, HVA responded to working environments, sleep disturbances, and isolation, VMA was associated with uncontrollable situations, and 5-HT was linked to anxiety. Both DA and HVA also responded to total stress severities, indicating that the "stress index" generated from the six urinary biomarkers could be a promising indicator to determine overall stress severities. "
Limitations	19	Discuss limitations of the study, taking into account sources of potential bias or imprecision. Discuss both direction and magnitude of any potential bias	14	"Several limitations of this study should be acknowledged. Firstly, the influence of sampling timing on urinary neurotransmitter levels needs to be assessed. Secondly, due to the limited impact of mental stress on urinary neurotransmitter changes, a larger sample size may yield more definitive outcomes. Thirdly, the effectiveness of early interventions, such as improving the working environment or removing stress factors, needs to be assessed based on empirical evidence. Lastly, while neurotransmitters are primarily synthesized in the brain, they are also produced in other organs such as the kidneys or gut."
Interpretation	20	Give a cautious overall interpretation of results considering objectives, limitations, multiplicity of analyses, results from similar studies, and other relevant evidence	14	"While neurotransmitters are primarily synthesized in the brain, they are also produced in other organs such as the kidneys or gut. Additionally, most neurotransmitters cannot pass through the blood-brain barrier, suggesting that the roles of neurotransmitters in the brain and other organs are independent. Therefore, it is crucial to investigate and clarify the underlying reasons why urinary neurotransmitters can serve as indicators of an individual's mental state."
Generalisability	21	Discuss the generalisability (external validity) of the study results	15	"This may provide clues for scientists to develop new screening tests for mental stress or depression. Further research and validation are required to establish the utility and accuracy of urinary neurotransmitter analysis as a diagnostic tool for mental stress."
**Other information**				

Funding	22	Give the source of funding and the role of the funders for the present study and, if applicable, for the original study on which the present article is based	-	This work was conducted with the support of our company, LSI Medience Corporation. The research did not receive specific grants from public or not-for-profit funding agencies.

*Give information separately for cases and controls in case-control studies and, if applicable, for exposed and unexposed groups in cohort and cross-sectional studies.

Note: An Explanation and Elaboration article discusses each checklist item and gives methodological background and published examples of transparent reporting. The STROBE checklist is best used in conjunction with this article (freely available on the Web sites of PLoS Medicine at http://www.plosmedicine.org/, Annals of Internal Medicine at http://www.annals.org/, and Epidemiology at http://www.epidem.com/). Information on the STROBE Initiative is available at www.strobe-statement.org.
